# SatTCR: a pipeline for performing saturation analysis of the T cell receptor repertoire and a case study of a healthy canine

**DOI:** 10.1016/j.mex.2025.103733

**Published:** 2025-11-27

**Authors:** Rene Welch Schwartz, Cindy L. Zuleger, Michael A. Newton, David M. Vail, Mark R. Albertini, Irene M. Ong

**Affiliations:** a Department of Biostatistics and Medical Informatics, School of Medicine and Public Health, University of Wisconsin-Madison, Madison, WI, USA; b Carbone Cancer Center, University of Wisconsin-Madison, Madison, WI, USA; c Department of Medicine, School of Medicine and Public Health, University of Wisconsin-Madison, Madison, WI, USA; d Department of Medical Sciences, School of Veterinary Medicine, University of Wisconsin-Madison, Madison, WI, USA; e Department of Dermatology, School of Medicine and Public Health, University of Wisconsin-Madison, Madison, WI, USA; f The Medical Service, William S. Middleton Memorial Veterans Hospital, Madison, WI, USA; g Department of Obstetrics and Gynecology, School of Medicine and Public Health, University of Wisconsin-Madison, Madison, WI, USA; h Center for Human Genomics and Precision Medicine, University of Wisconsin-Madison, Madison, WI, USA

**Keywords:** Canine, T cell receptor beta chain, Immune profiling, Illumina sequencing, Comparative oncology, Saturation analysis

## Abstract

**Motivation:**

Profiling the T cell receptor (TCR) repertoire using next-generation sequencing (NGS) to quantify adaptive immune responses has become common in human and animal research. Companion dogs with spontaneous tumors have similarities with humans who have cancer. T cells undergo clonal expansion when they recognize specific antigens via surface TCRs. TCR counts from NGS data provide a way to quantify T cell response to vaccines, cancer, or infectious diseases for preclinical and clinical health studies. One complication is that the power and accuracy of TCR experiments depend substantially on the TCR sequencing depth, therefore it is important to determine the optimal read depth of an experiment to verify whether a subject’s repertoire is correctly represented.

**Results:**

The optimal TCR sequencing depth for future experiments can be determined by randomly sampling lower TCR sequencing depths from a sequencing experiment, assembling the TCR clonotypes, and determining where the saturation of power and accuracy occurs. Moreover, one can determine whether an existing experiment has sufficient sequencing depth to justify its conclusions. We provide guidelines to determine whether the sequencing depth is adequate and a computational pipeline that:

Samples pairs of sequences and assembles clonotypes

Summarizes the results in a parametrized report

## Graphical abstract

**Figure:**
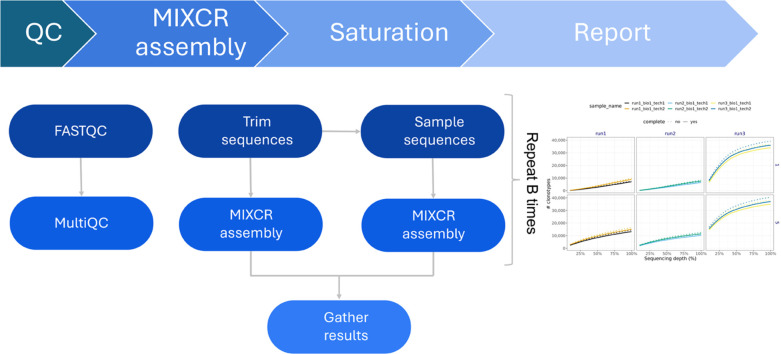

Specifications table

**Table:** 

**Subject area**	Bioinformatics
**More specific subject area**	System Immunology
**Name of your method**	SatTCR
**Name and reference of original method**	MiXCR Dmitriy A. Bolotin, Stanislav Poslavsky, Igor Mitrophanov, Mikhail Shugay, Ilgar Z. Mamedov, Ekaterina V. Putintseva, and Dmitriy M. Chudakov. 2015. “MiXCR: Software for Comprehensive Adaptive Immunity Profiling.” *Nature Methods* 12 (5): 380–81
**Resource availability**	https://github.com/Ong-Research/SatTCR

## Background

Profiling the TCR repertoire using NGS has become common in translational research. T cells play an essential role in the adaptive immune system and the TCR repertoire is a record of the individual’s lifetime immune response history. T cell specificity is determined by a cell-surface antigen receptor termed the T cell receptor [1]. Analysis of the TCR repertoire as a tool to understand immunological responses to cancer in humans has been widely studied [[2], [3], [4], [5]].

The companion canine parallel population’s role as a valuable model for human disease, including allergies, autoimmune disorders, and cancer has entered the mainstream discourse [6]. Rodent cancer models have been essential for the mechanistic study of immune therapies, but these models are limited when predicting the effectiveness of immune therapies in humans [7]. In contrast, companion canine parallel populations, whose immune exposures are more similar to humans, would improve our understanding of the biology and treatment effects in human cancer due to the similarities with human populations in histology, biologic behavior, and molecular abnormalities [8]. Dogs are a large and genetically diverse out-bred population and share matching environmental exposures with humans. This is especially true for melanoma, as dogs also develop malignant melanoma spontaneously in the setting of an intact immune system [9].

Multiple methods exist to generate TCR repertoire data, and the advantages of each method have been characterized in humans and mice [10]. Profiling methods for TCR repertoire data have been thoroughly reviewed [11]. Comparison of MIXCR [12,13] processing of TCR data generated by Rapid Amplification of cDNA Ends (RACE) with multiplex Polymerase Chain Reaction (PCR) protocols, MIXCR’s approach has been demonstrated to be optimal for detecting maximal diversity of clonotypes [14]. There is limited TCR data specific to the canine repertoire, and to the best of our knowledge only for B cell receptor (BCR) repertoire a computational pipeline specific for the canine genome has been developed [15]. Recent studies have explored single cell TCR data to profile the canine TCR repertoire [16,17], however, using single cell sequencing methods to perform population studies would be prohibitive as the number of subjects increase. Here we provide guidelines for the computational analysis of canine TCR repertoire by describing an in-house developed protocol [18]

## Method details

### Study details, sample collection, and sample preparation

This study was approved by the Institutional Animal Care and Use Committees of the University of Wisconsin School of Veterinary Medicine (protocol V006037) and the William S. Middleton Memorial VA Hospital (protocol MRA0001). All experiments conformed to the relevant regulatory standards. Written informed consent was obtained from the companion dog’s caregiver before study entry. The data presented herein are from peripheral blood collected from a single healthy dog receiving care at the University of Wisconsin Veterinary Care. Peripheral blood mononuclear cells (PBMC) were cryopreserved in multiple vials and stored at −140 °C. Clustal Omega (1.2.4) [19] was used to align exon 1 of canine TRBC1 and TRBC2 (data not shown). The samples were prepared as previously described [18].

### Sequence trimming

We trimmed the raw sequence data using trimmomatic (0.39) [20] using the `TRAILING:20` and `MINLEN:100` parameters to remove bases with poor quality reads. This configuration removes the trailing nucleotides of the sequences when the phred quality score is less than or equal to 20, then it removes sequences that are <100 base pairs (bps) in length.

### T cell receptor repertoire assembly

We utilized MIXCR (4.1.2) [12] to assemble the TCR repertoire using the International ImMunoGeneTics information system (IMGT) [21] TCR beta chain (TRB) sequences for the canine genome. MIXCR aligns the sequences to the IMGT’s database containing V and J gene sequences, which comprise the TCR gene, and archives high gene assignment accuracy for both gene families. Next, MIXCR assembles identical and homologous reads into clonotypes and corrects for PCR and sequencing errors. MIXCR’s output report consists of a row for each clonotype with columns: id, abundance, V gene, D gene, J gene, C gene, Complimentary determining region 3 (CDR3) nucleotide sequence, and CDR3 amino acid sequence. We studied clonotypes in the TRB and defined a pair of clonotypes to be the same if they were annotated by the same V and J genes and presented the same CDR3 nucleotide sequence [22].

### Diversity

We calculated the following diversity metrics: The total number of clonotypes (n), the Shannon diversity (H), and the D50 diversity indexes [23,24] given by:

• $H=-\sum_{j=1}^{n}p_{j}\;\log\;p_{j}$ where $p_{j}$ is the frequency of copies in the repertoire for the j-th clonotype.
• $D50=\min\{k:\sum_{j=1}^{n-k+1}p_{\left(n-j+1\right)}\geq 0.5\}$ where $p_{\left(1\right)}\leq p_{\left(2\right)}\leq \cdots \leq p_{\left(n\right)}$ are the order statistics of the clonotype frequencies. The D50 index is the rank required across the clonotype frequencies to accumulate 50 % of all the repeated sequences after sorting them by frequency in descending order.

### Saturation

We used a sequential bootstrap approach [25] to randomly sample blocks of trimmed paired sequences, where we first divided the paired sequences into blocks of approximately 10 % of the total amount of sequence pairs and then aggregated them to have samples with increasing sequencing depth such that the larger samples contain the smaller samples. Finally, we assembled the clonotypes using MIXCR as above and repeated the complete procedure B times (by default *B* = 10). The saturation figures show the loess [26] curves of each statistic as a function of the sampled sequencing depth.

### Principal coordinate analysis

We used powerTCR (1.22) [27] to fit a biologically interpretable model to the TCR distribution of each sample separately. Next, we compared the samples by calculating the JSD between the fitted probabilistic models for each pair of samples, defined by:

• JSD(P∥Q)=0.5×D(P∥M)+0.5×(Q∥M)
• $D\left(P∥Q\right)=\sum_{i=1}^{n}p_{i}\log\left(p_{i}/q_{i}\right)$

where M=0.5×(P+Q), and P,Q are vectors of probabilities with n elements each. This allowed us to compute the principal coordinate components and evaluate whether the samples are separated by sequencing depth by using a PERMANOVA [28] test over the distance matrix using the vegan (2.6–4) [29] R package by considering the run as a dependent variable and using 10 K permutations.

### Other quantitative analysis

Visualization of TCR data was generated using the R packages ggplot2 (3.4.4) [30], and ComplexUpSet (1.3.5) [31,32].

### SatTCR pipeline usage

The SatTCR pipeline is publicly available in GitHub at https://github.com/Ong-Research/SatTCR. The source code is available in the repository, and there is an accompanying website at https://ong-research.github.io/SatTCR/ with detailed information on how to run the pipeline and interpret the results, and it is designed to be operated through Docker containers.

I. Setting up the SatTCR pipeline to analyze TCR repertoire data:

• The SatTCR is publicly available in GitHub at https://github.com/Ong-Research/SatTCR, and to download the pipeline there are two alternatives:
  • Download from the command line using the command:
    `git clone git@github.com:Ong-Research/SatTCR.git`
  • Manually download the repository from a released link.
• The SatTCR pipeline ensures reproducibility by using Docker [33] containers and Snakemake [34], and to install them, the instructions are available in their sites:
  • Docker: https://www.docker.com/
  • Snakemake: https://snakemake.readthedocs.io/en/stable/
• Pull Docker images that are going to be utilized by the pipeline, using the commands after changing to the SatTCR directory using `cd SatTCR`
  • FastQC: `docker pull staphb/fastqc`
  • MultiQC [35]: `docker pull staphb/multiqc`
  • Trimmomatic [20]: `docker pull staphb/trimmomatic`.
  • R / Quarto: `docker build -t tcr/sattcr - < Dockerfile`
  • MIXCR: `docker pull ghcr.io/milaboratory/mixcr/mixcr:latest`, additional MIXCR images are available in https://github.com/milaboratory/mixcr/pkgs/container/mixcr%2Fmixcr
• Get MIXCR license from https://mixcr.com/mixcr/getting-started/milm/ and save it into a file.
• Optionally, download and unzip the IMGT [21] library release.
• Create a comma-separated value (csv) with 2 columns:
• `sample_name`: The name of the sample.
• `sample_file`: The prefix of the files until before the `_R1` and `_R2` parts, e.g. if the pair of RNA-seq files are data/sample1_R1_L001.fastq.gz and data/sample1_R2_L001.fastq.gz, then this column is `data/sample1`.
• Edit the `config/config.yaml` file. This file is divided by pieces in order to easily configure running the pipeline.
• General configuration parameters:
• `threads`: Max. # of parallel threads used per process.
• `samplefile`: Location of the file with the samples
• `seed`: Seed number for random number generation and sequence sampling during saturation analysis.
• `run_*`: Logical indicators to determine if running a stage of the pipeline
• `suffix`: This is regarding to the `samplefile`. If the pair of RNA-seq files are `data/sample1_R1_L001.fastq.gz` and `data/sample1_R2_L001.fastq.gz`. The suffix would be the remaining part after the R1/R2 parts, i.e. `_L001.fastq.gz`.
• Docker configuration parameters:
• `run_line`: This is the docker command used to run every rule.
• `fastqc`, `multiqc`, `trimmomatic`, `rquarto` and `mixcr` are the names of the images that were pulled before.
• In general, it is not necessary to modify these parameters unless a different image name is used or a specific need to configure how docker runs in the user’s system.
• Trimmomatic configuration parameters:
• `trimmer`: A vector with the `trimmomatic` configuration to use. More information is available in http://www.usadellab.org/cms/?page=trimmomatic. But the general idea is to remove the low-quality nucleotides at the end of the sequences, or very short sequences.
• MIXCR configuration parameters:
• `params`: The configuration line used to control MIXCR behavior. We used the line `rna-seq –species dog -b imgt.202214–2 –rna`, and `rna-seq –species has -b imgt.202214–2 –rna` to assemble the data from Zuleger et al. 2023 [18] and Zuleger et al. 2020 [36], respectively. MIXCR provides a comprehensive list of preset configurations in https://mixcr.com/mixcr/reference/overview-built-in-presets/.
• `license_file`: Location of the file with the license. The pipeline uses this file to run MIXCR in a docker container.
• Saturation configuration parameters:
• `samples`: A vector with the sample keys for which the saturation analysis is going to be processed
• `block_size` or `nblocks`: Either the # of sequences that are going to be sampled by block or the # of blocks of sequences used to split the original sequence files.
• `bootstrap_replicates`: The number of times that the block bootstrap sampling procedure is going to be repeated.
  This rule is computationally intensive, because in total there are going to be sampled $\left(n_{blocks}-1\right)\times n_{bootstrap\;replicates}$ pairs of sequence files and then MIXCR is used for each pair of files.

II. Running the pipeline

In the instructions below, the flag `-c{k}` stands for running the rule with {k} parallel threads.

1) Quality control: `snakemake -c{k} qc`. The output of this rule is a html report generated with MultiQC and quality profiles generated with the R package dada2 [37]. Either one of these analyses will depict quality score summaries at each position of the sequence files.
2) Trim sequences: `snakemake -c{k} trim`. The output of this rule are the trimmed versions for every raw sequence file.
3) Clonotype assembly with MIXCR: `snakemake -c{k} mixcr`. The output of this rule is a tsv file according to the AIRR format (https://docs.airr-community.org/en/stable/datarep/overview.html) for every set of RNA-seq paired files.
4) Block bootstrap sampling: `snakemake -c{k} sampling`. This rule generates $\left(n_{blocks}-1\right)\times n_{bootstrap\;replicates}$ pairs of compressed fastq files.
5) Saturation analysis: `snakemake -c{k} saturation`
6) Generate the report: `snakemake -c{k} report`. This rule produces an html report compiled by `quarto` summarizing the results of the analysis.

III. Examination of the results

• The report section presents summary statistics for each sample and their TCR repertoire, a detailed analysis of the clonotypes assembled by MIXCR and a detailed saturation analysis (Supplementary Figure 1). Example reports of the SatTCR pipeline are available in https://ong-research.github.io/SatTCR/cases/canine_tcr/ and https://ong-research.github.io/SatTCR/cases/zuleger_2020/. The first report above is a reproduction of the analysis in this manuscript, and the second illustrates the pipeline using human samples from Zuleger et al., 2020 [36]. Fig. 2 illustrates the key aspects when examining the SatTCR pipeline report.

### Method validation

Using the data generated by Zuleger et al., 2023 [18] we analyzed two scenarios of immunological relevance, one with a high sequencing depth representing deep sampling and one with a low sequencing depth representing a budget-oriented sampling of the canine TCR repertoire. Each run is composed of two different biological replicates, and within each of them, two technical replicates were sequenced, showing robust and reproducible detection of canine TRB (Supplementary Figure 2).

We assembled the clonotypes by processing all samples using MIXCR and summarized statistics for each sample (Table 1). The sequencing depth for sequencing runs 1 and 2 were very similar across the biological replicates Bio1 and Bio2 and across the technical replicates Tech1 and Tech2. As expected, samples with larger sequencing depth yield more clonotypes, but the increase in the number of clonotypes is not proportional to the increase in sequencing depth because the run1 and run 2 samples do not completely capture the clonotypes in the canine TCR repertoire. Then, we examined the clonotype overlap between the biological and technical replicates of each run and noticed that the majority of the clonotypes were replicate-specific. Moreover, the clonotypes that are present in more than one replicate usually are repeated more often than the replicate-specific clonotypes, where the effective average counts are the highest in average when corresponding to the clonotypes assembled by all replicates (Supplementary Figure 3, data for run 2 not shown). In summary, replicate sequencing enables reliable calling of TCR clonotypes because the clonotypes assembled by more than one replicate independently are repeated more often.

**Table 1:** Summary statistics per sample. There are 3 runs and 4 replicates per run. The columns are sample name, sequencing depth, # of assembled clonotypes, the number of clonotypes with a minimum abundance of 5 copies, the ratio between both quantities, the Shannon diversity, and the D50 indexes.

Sample name	Seq. depth	# clonotypes (count ≥ 1)	# clonotypes (count ≥ 5)	Ratio	Shannon diversity	D50 index
run1_bio1_tech1	364,450	13,113	7047	53.73 %	8.22	1060
run1_bio1_tech2	470,981	14,884	8935	60.03 %	8.32	1170
run1_bio2_tech1	352,987	15,562	7183	46.15 %	8.42	1229
run1_bio2_tech2	318,514	16,989	6496	38.23 %	8.45	1333
run2_bio1_tech1	389,927	10,096	6475	64.13 %	7.92	767
run2_bio1_tech2	472,274	11,345	7629	67.24 %	8.03	859
run2_bio2_tech1	376,741	12,896	7409	57.45 %	8.22	1001
run2_bio2_tech2	341,417	13,935	7392	53.04 %	8.27	1102
run3_bio1_tech1	2728,331	34,360	33,670	97.99 %	9.31	3496
run3_bio1_tech2	3357,039	36,375	35,712	98.17 %	9.35	3643
run3_bio2_tech1	2.605,340	39,455	38,655	97.97 %	9.48	4299
run3_bio2_tech2	2485,771	41,488	40,600	97.85 %	9.51	4796

**Fig. 1:**
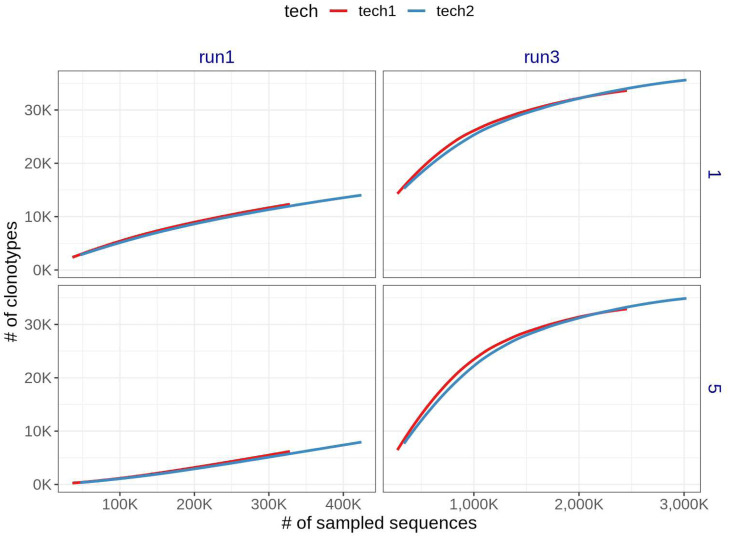
Saturation analysis for two technical replicates of the same sample. The x-axis is the # of sampled sequences and the y-axis is the # of clonotypes assembled. The colors correspond to the technical replicates of the bio1 samples, where the vertical panels correspond to the lower (left) and higher (right) sequencing depths. The horizontal panels correspond to the minimum abundance to consider a clonotype.

We then asked, what would be an appropriate sequencing depth for TCR samples in the canine genome. By sampling blocks of increasing sequencing depth, we observed that when the sequencing depth from the original depth is shallow, then the trend between increments of sampled sequences and clonotypes looks approximately linear. However, in the opposite scenario, we notice a concave pattern which indicates that the probability of observing a new clonotype when increasing the number of sequences becomes lower as the sequencing depth increases (Fig. 1, data for run 2 not shown). These patterns are consistent with theoretical predictions on the relationship between sample diversity and sample size, for instance in discrete-population sampling via the Pitman-Yor process [38,39].

**Fig. 2:**
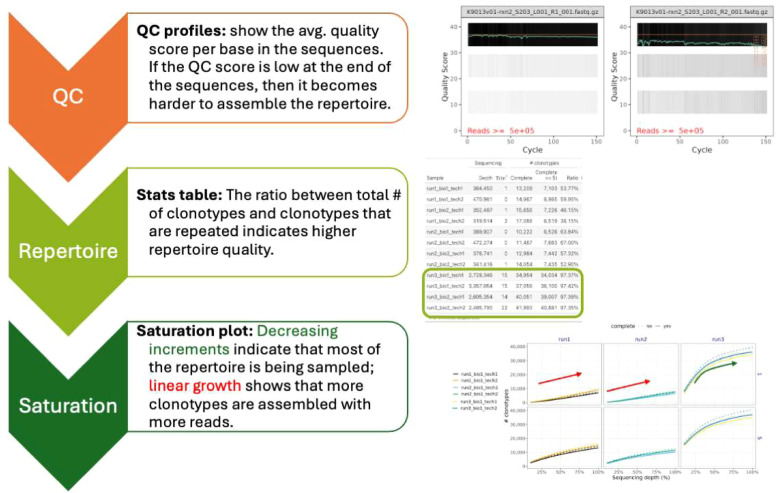
Report interpretation guidelines. Diagram with key aspects to examine when looking at SatTCR report.

Finally, we compared how all samples relate to each other. We used powerTCR to fit a probabilistic model to the count distribution for each sample separately. Supplementary Figure 4A reveals that this model accurately fits the abundance distribution for each sample. Next, utilizing the JSD between the fitted models as a distance we computed the principal coordinate components, the left and middle panels of Supplementary Figure 4B depict the first component representing a clear contrast between Run 3 and the remaining samples. We confirmed that the main source of variability between the samples is the sequencing depth by using a PERMANOVA test (p.value = 0.0025).

By studying the canine TCR repertoire we confirmed that a higher sequencing depth is required to capture a correct identification of every representation. Computationally, we adapted previously established methods for studying the sequencing depth of a repertoire experiment [40] and determined that with between 2.5 million (M) and 3 M sequencing reads it is possible to capture a correct representation of the canine TCR repertoire. However, using 1 M reads yields approximately 60 % of the clonotypes assembled with >2.5 M sequencing reads. We adapted ecological methods to show that as expected the sequencing depth is one, if not the major, source of variation between different replicates of the same sample. Moreover, we illustrated that replicate sequencing allows for better control of called clonotypes.

Deep sequencing multiple replicates could be expensive, but we consider that the benefits of this practice outweigh the costs because it enables the reliable assembly of TCR clonotypes. To more completely profile a subject’s TCR repertoire, we suggest that canine TCR repertoire samples have >1.5 M sequence pairs and at least sequence technical replicates. However, sequencing biological replicates instead of, or in addition to, technical replicates may offer superior sensitivity as signals or patterns revealed in multiple biological replicates are more likely to be due to true biologic signals. However, if the experiment’s objective is to study the VJ gene pairings, determining whether an expanded clone is present in the repertoire or to compute a statistic that summarizes the repertoire such as the Shannon or D50 diversity indexes, then using 300 K sequence pairs may be an appropriate consideration

## Limitations

A limitation of our study is the fact that we only present RNA data from one dog. However, we analyzed both biological and technical replicates and the data show a robust and reproducible detection of the canine TRB, and we also reference and illustrate an example of results for a prior study with human samples [36]

## Ethics statements

The experiments complied with the ARRIVE guidelines and were carried out in accordance with the U.K. Animals (Scientific Procedures) Act, 1986 and associated guidelines; EU Directive 2010/63/EU for animal experiments; or the National Institutes of Health guide for the care and use of laboratory animals (NIH Publications No 8023, revised 1978)

## CRediT authorship contribution statement

**Rene Welch Schwartz:** Methodology, Software, Formal analysis, Data curation, Writing – original draft. **Cindy L. Zuleger:** Methodology, Conceptualization, Investigation, Writing – review & editing. **Michael A. Newton:** Formal analysis. **David M. Vail:** Methodology, Conceptualization, Investigation, Writing – review & editing, Supervision, Funding acquisition. **Mark R. Albertini:** Formal analysis. **Irene M. Ong:** Methodology, Conceptualization, Software, Formal analysis, Writing – original draft, Supervision.

## Declaration of competing interest

The authors declare that they have no known competing financial interests or personal relationships that could have appeared to influence the work reported in this paper.

## Data Availability

I have shared the link to my data/code at the Attach file step

## Appendix Supplementary materials

Image, application 1
