# Supplementary material for: SatTCR: a pipeline for performing saturation analysis of the T cell receptor repertoire and a case study of a healthy canine
Source: MethodsX. 2025 Nov 27;16:103733. doi: 10.1016/j.mex.2025.103733 (PMC12743510; doi:10.1016/j.mex.2025.103733)
Supplement: Supplementary file 1 [file mmc1.docx]

**Supplementary Figure 1**

**SATCR report example.** Screenshot of the report generated by SATCR.


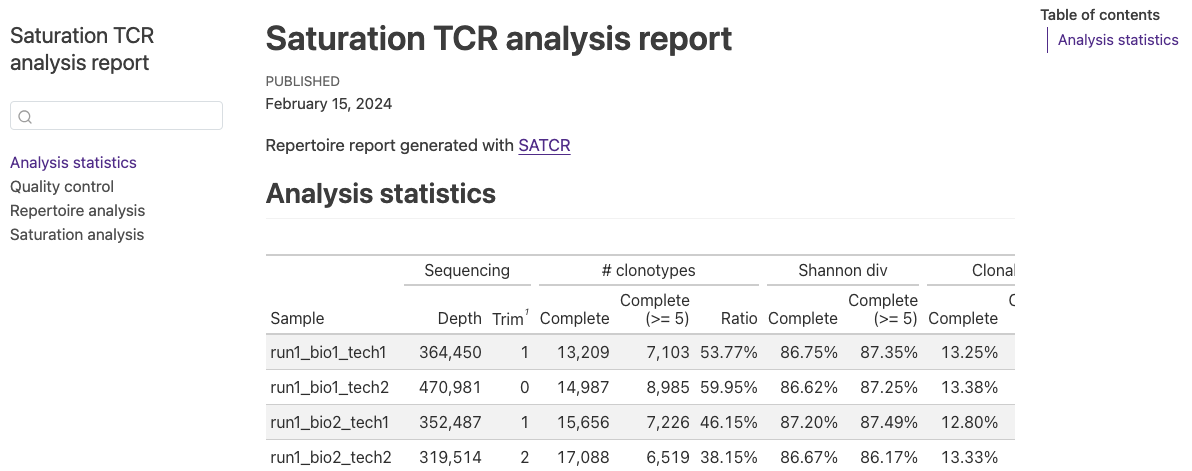


**Supplementary Figure 2**

**VJ gene usage.** Percentage of clonotypes annotated by a V (top) and J (bottom) gene stratified by replicate (biological and technical).


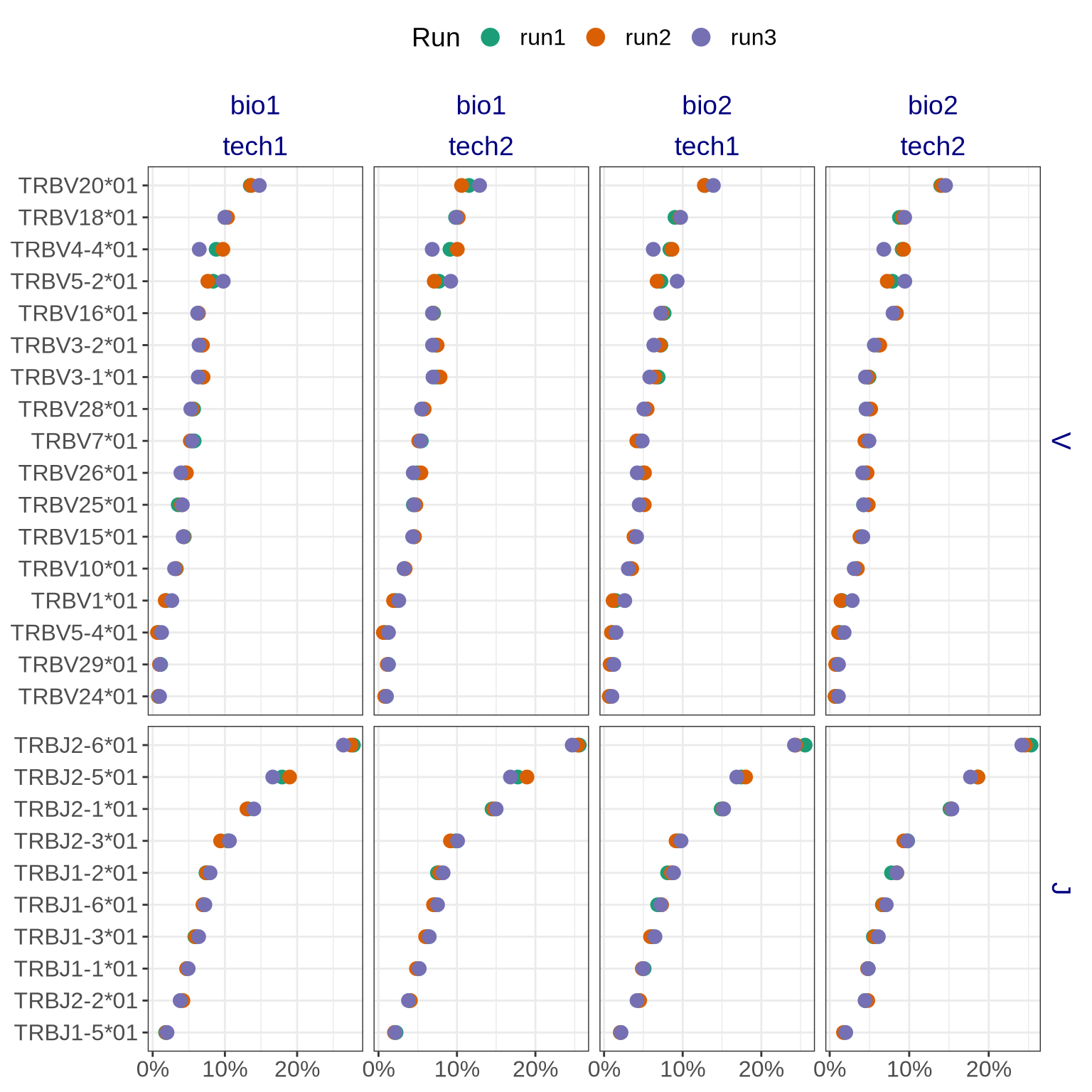


**Supplementary Figure 3**

**UpSet plot comparing 4 replicates for two runs, and scatter plots comparing log(count) values for the replicates.** UpSet plots for run1 (top) and run3 (bottom). The top panel shows the intersection size of the 4 replicates highlighted in the bottom panel. The top middle panel shows the percentages of clonotypes in the intersection stratified by count ranges. The bottom middle panel compares the effective average count defined as the average of the samples present in the intersection. The bottom panel highlights the intersection that is considered in the top panels.

A)
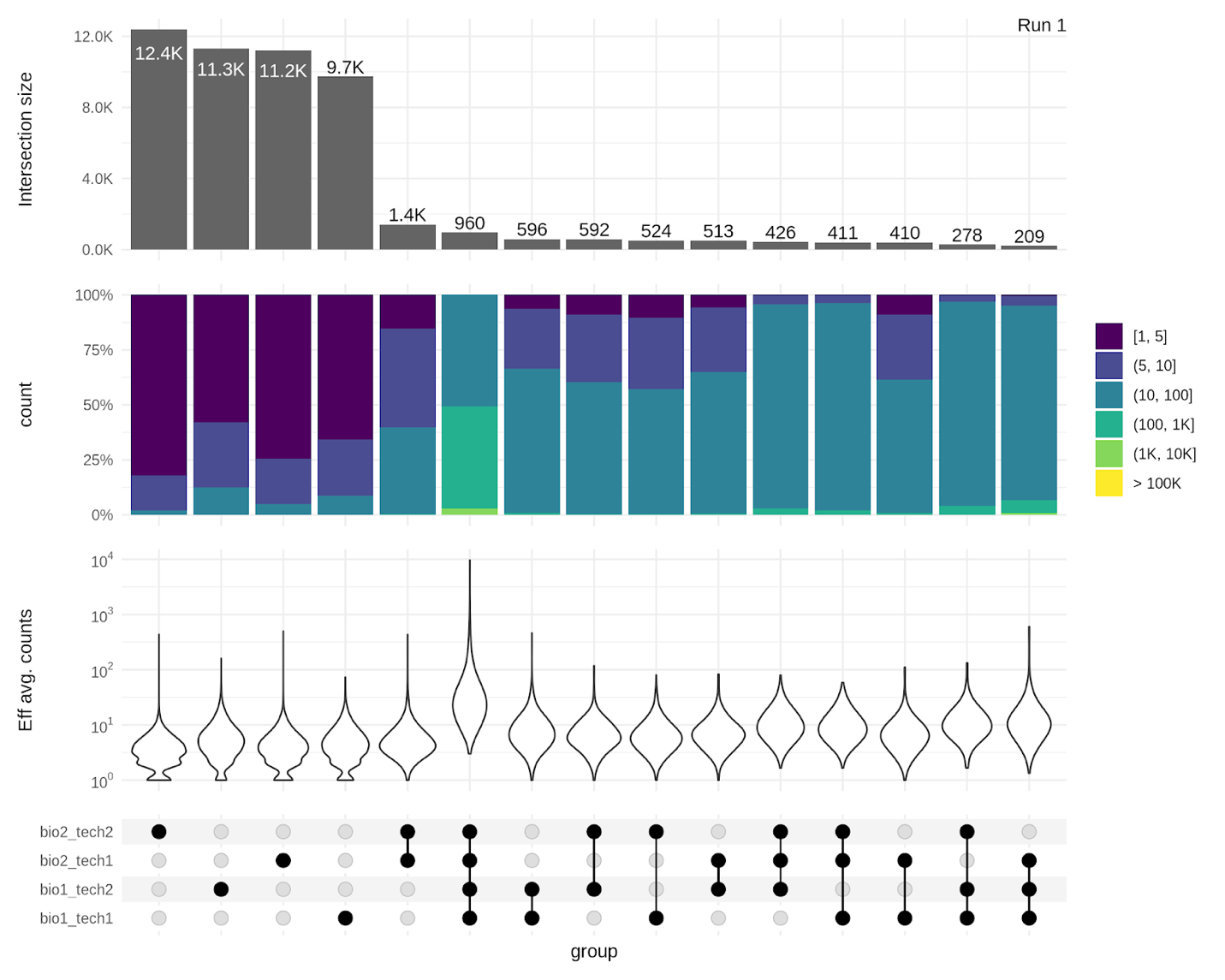


B)

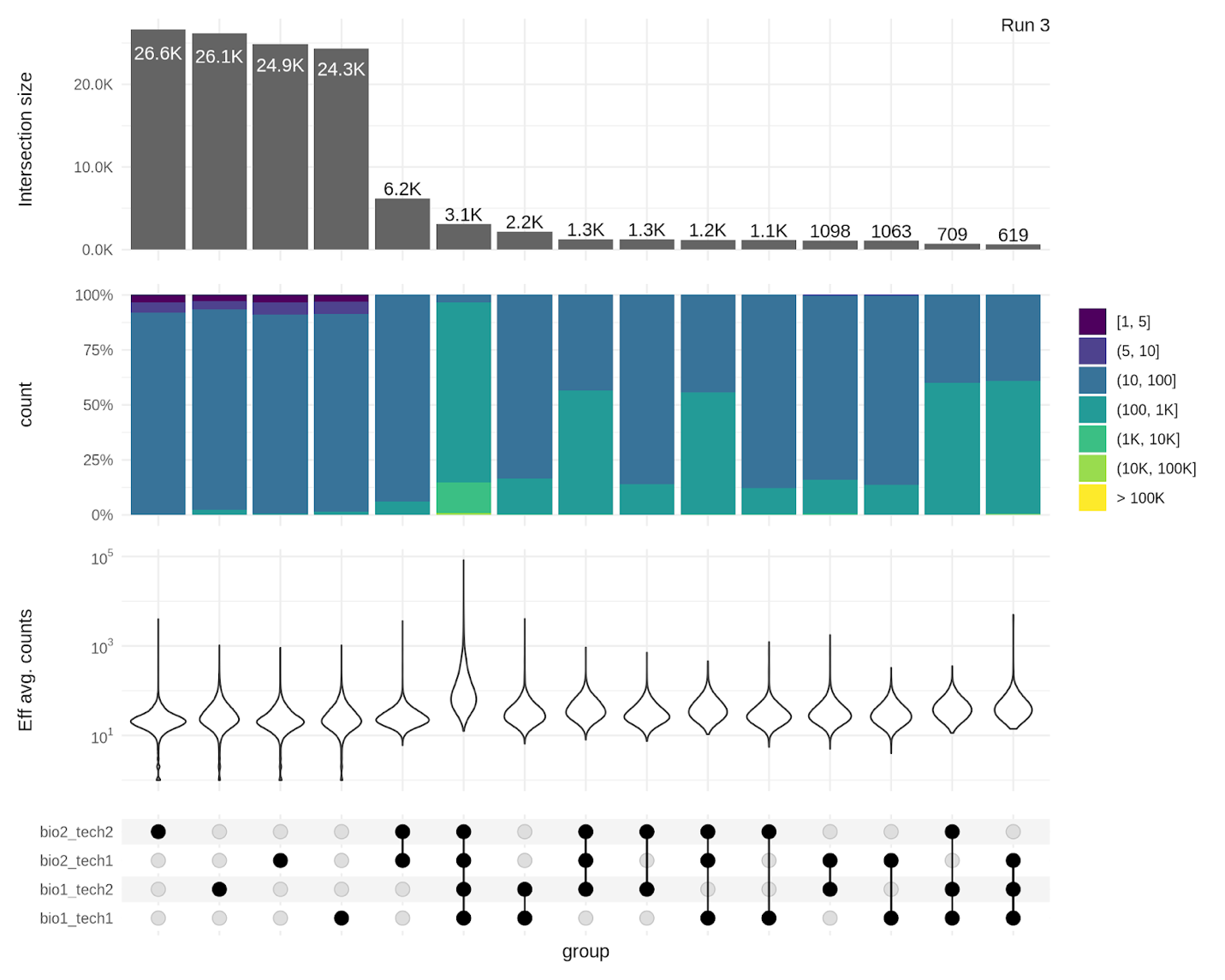


**Supplementary Figure 4**

**Principal coordinate analysis.** The top figure shows the fitted models using powerTCR. The bottom is a comparison of the 12 samples across the top 3 principal coordinate components colored by biological and technical replicates per run.

A)


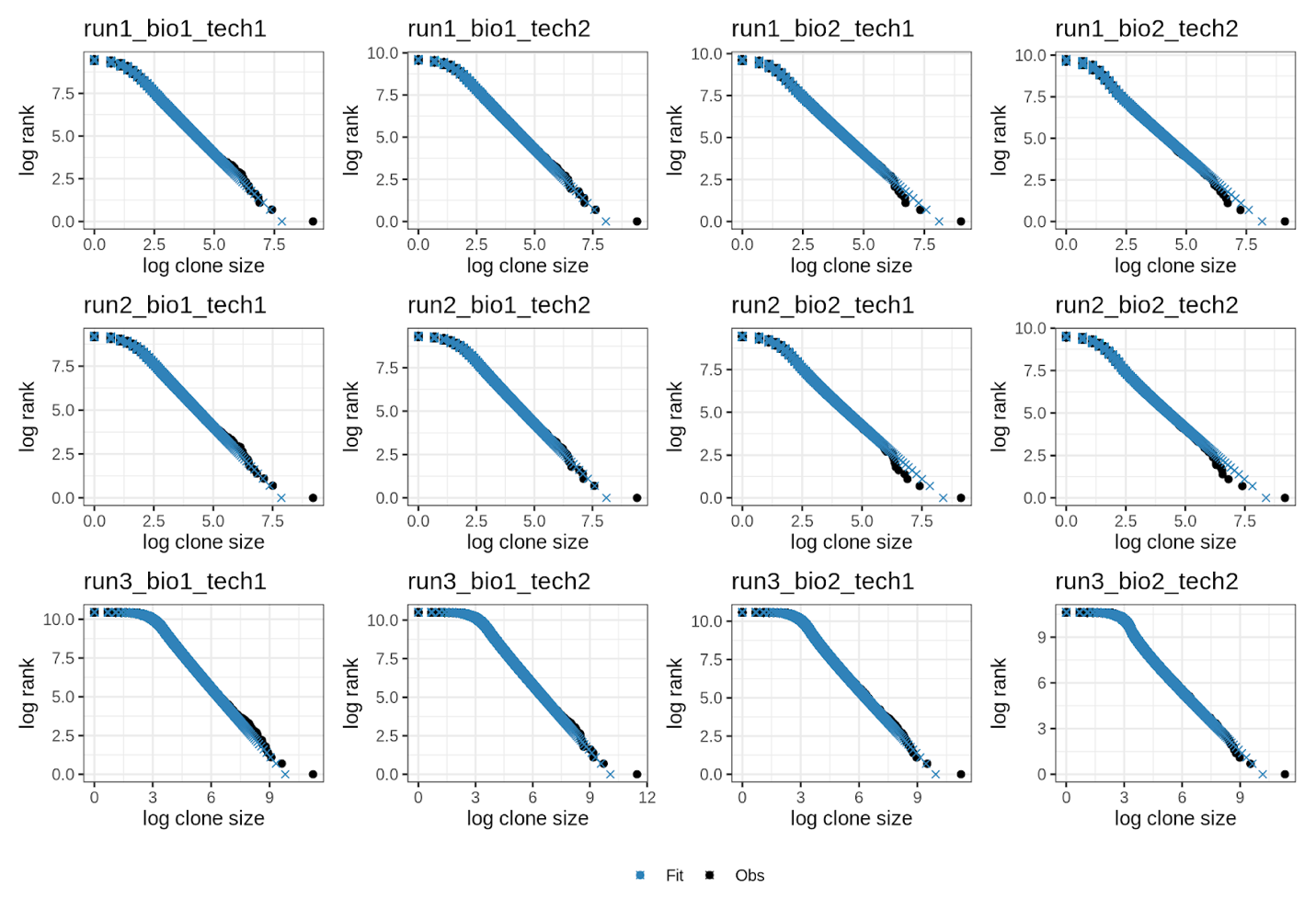


B) **
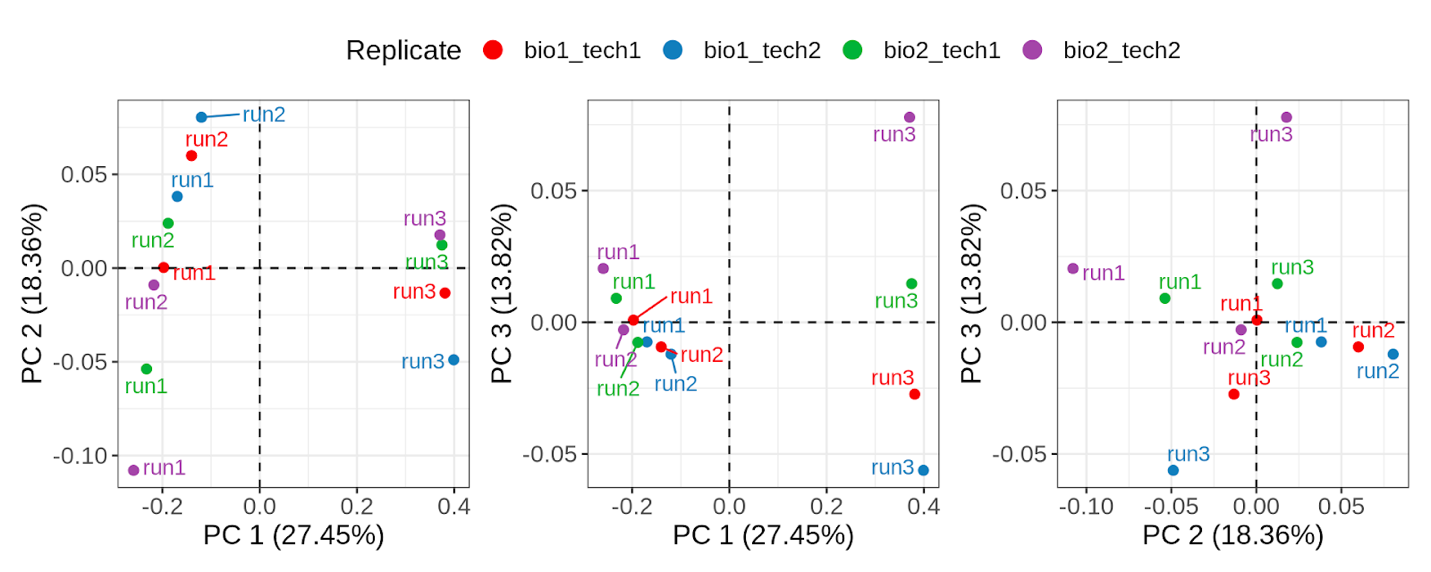
**
